# California mortality and the Healthy Places Index

**DOI:** 10.1093/aje/kwae418

**Published:** 2024-11-14

**Authors:** Neil Maizlish, Adrienne Damicis

**Affiliations:** Public Health Alliance of Southern California, Public Health Institute, Oakland, CA, United States; Public Health Alliance of Southern California, Public Health Institute, Oakland, CA, United States

**Keywords:** social determinants of health, mortality, COVID-19, deprivation index, health equity, racial equity, area-based socioeconomic measure

## Abstract

We investigated California mortality and social determinants of health, as measured by the Healthy Places Index (HPI), which is a composite measure of 23 indicators of neighborhood (census tract) economic conditions, education, transportation, housing, social capital, environmental pollution, built environment, and access to health care. We aggregated deaths to 2010 census tract boundaries for leading causes during 2015 to 2019, and for COVID-19 during 2020-2021, from death certificates, and populations from the American Community Survey, 2015 to 2019. We age-adjusted and stratified death rates by HPI deciles, age, sex, and race/ethnicity, and examined HPI dose-response with segmental regression. For all causes, cancer, cardiovascular disease, COVID-19, diabetes, cirrhosis of the liver, injuries, and Alzheimer’s disease (ages 65-74 years), mortality rates were inversely related to HPI decile. For all-cause mortality, the rate ratio between the 1st and 10th deciles (reference) was 1.63 (95% CI, 1.60-1.66); for COVID-19, the rate ratio was 7.61 (95% CI, 7.14-8.12). The population attributable fraction was 24% for all causes and 72% for COVID-19. Age, sex, and race/ethnicity modified the steepness and shape of dose-response curves. The findings illustrate opportunities to incorporate area-based socioeconomic measures in routine public health surveillance and to reinforce policies and programs that reduce health inequities.

## Introduction

Racial and socioeconomic health inequities were a defining characteristic of the COVID-19 pandemic in the United States.^1^ To quantify and monitor COVID-19 health disparities, federal and state health authorities incorporated area-based socioeconomic measures into public health surveillance.^2^^-^^4^ This approach was integrated into the pandemic response in California, which adopted the California Healthy Places Index (HPI)^2^^,^^5^ as a health-equity metric for rate-based surveillance of COVID-19 cases, test positivity, deaths, and vaccine coverage.

The California Department of Public Health reported a linear, inverse relationship between COVID-19 mortality in 2020 and HPI quartiles that was independent of age, race/ethnicity, sex, and occupation/industry.^6^ We investigated whether the social gradient in HPI-associated COVID-19 deaths was generalizable to prepandemic causes of death, and whether age, sex, and race/ethnicity were effect modifiers of dose response. We also investigated whether a more finely stratified HPI scale using deciles reveals a dose-response with practical implications for California policymakers, who prioritize populations in census tracts with the lowest 25% of HPI scores (ie, least healthy community conditions). In this article, we summarize and interpret a selection of analyses compiled in a technical report.^7^

## Methods

### Health and health equity outcomes

The principal outcome was the mortality rate of underlying cause of death for major diagnostic categories of the Global Burden of Disease as modified by the California Department of Public Health.^8^ Underlying cause of death was classified according to guidelines of the National Center for Health Statistics and was coded to the *International Classification of Diseases, 10th Revision* (ICD-10). COVID-19 was classified by the ICD-10 code U07.1. Because COVID-19 significantly affected the pattern of deaths, we examined deaths in 2 time periods: 1) 2015-2019 (pre–COVID-19) and 2) 2020-2021 (pandemic peak). For a specific cause of death, *i*, over *n* years, the cause-specific mortality rate (*MR*), was defined as: ${MR}_i=\left(\sum_1^n{deaths}_i/n\right)/P$, where *P* is the average annual population. The principal health equity outcome was the ratio of age-adjusted rates or age-, sex-, and race/ethnicity-stratified rates of the first through ninth deciles of the HPI with the 10th decile being the a priori reference for healthiest community conditions. We calculated attributable deaths within age groups by multiplying the difference in rates of the first 9 deciles and the 10th decile and respective decile populations and summing over age groups: $\sum_{j=1}^5\ \sum_{k=1}^{10}\left({MR}_{j,k}-{MR}_{j,10}\right)\times{P}_{j,k}$, where *k* is the k^th^ HPI decile and *j* is the j^th^ age group.

### Data sources

The California Comprehensive Master Death File recorded sex, birth and death dates for calculating age at death, race and ethnicity, and geographic identifiers such as residential address, and 2010 census tract from death certificates.^9^ The American Community Survey (ACS) was the source for annual average population, 2015 to 2019, in strata of age, sex, and race/ethnicity.^10^ No other source, including modeled Surveillance, Epidemiology, and End Results population data,^11^ was available for 2010 census tracts that allowed for disaggregation of race/ethnicity, particularly Asian and Native Hawaiian/Pacific Islander groups. The HPI scores for 2010 census tract boundaries and values of individual indicators are available from the Public Health Alliance of Southern California.^12^

### HPI score

The Public Health Alliance of Southern California (Public Health Institute, Oakland, CA) in partnership with Virgina Commonwealth University created the California HPI, version 3.0,^13^^,^^14^ which is a composite, additive score of 23 indicators of community conditions for California census tracts, 2015-2019 (Table 1). Eligible census tracts have a population of at least 1500 and a share of group-quarters population of less than 50%. The HPI indicators are produced from ACS and 7 other public sources^14^ and are grouped into 8 domains (called policy action areas) of social determinants of health (SDOH): economy, social, education, housing, transportation, pollution, neighborhood built environment, and health care access. The *z* scores of indicators within domains are averaged, and weighted quantile sums regression is used to assign domain weights based on the association between the overall HPI score and life expectancy at birth. The overall HPI score is the sum of the weighted domain average *z* scores. The methods have been previously described.^12^^,^^13^

**Table 1:** Census tract mean Healthy Places Index (HPI) indicator values at the 1st and 10th decile of the HPI score, 2015-2019, California.

**Indicators by domain (weight)**	**HPI decile**	
	**1st**	**10th**
Economic (35%)		
Individuals at >200% of federal poverty level, %	37	90
Employed, aged 20-64 years, %	62	79
Per capita income year^–1^ ($)	15 090	81 831
Education (18%)		
Adults aged ≥25 years with bachelor’s degree, %	9	69
Aged 15 to 17 years and enrolled in high school, %	95	99
Aged 3 to 5 years and enrolled in preschool, %	37	79
Social (13%)		
Registered voters voting in 2016 general election, %	60	88
Households responding to 2010 Census short form, %	56	80
Transportation (13%)		
Persons aged ≥16 years who walk, cycle, or commute by public transit, %	11	17
Households with access to a motor vehicle, %	85	94
Housing (5.3%)		
Households owning own home, %	32	69
Homeowners with severe housing cost burden, %	17	9
Renters with severe housing cost burden, %	33	16
Households with complete kitchen and plumbing, %	98	99
Households with < 1 occupants per room, %	79	98
Neighborhood (5.3%)		
Population with <0.5 mile access to a park, %	68	90
Retail density (jobs acre^−1^)	4.68	10.62
Area tree canopy, %	5	15
Environment (5.3%)		
Diesel particulate matter (μg m^−3^)	0.32	0.20
Fine particulate matter, PM_2.5_ (μg m^−3^)	11.3	9.08
Ozone, ppm	0.05	0.04
Water contaminants score	606	317
Health care access (5.3%)	79	97
Adults aged 18 to 64 years with health insurance, %		

Abbreviations: HPI, Healthy Places Index; PM_2.5_, airborne particulate matter with a diameter ≤2.5 microns.

By geocoding residential address and linking to census tract, each decedent was assigned an HPI score. Likewise, using ACS 5-year, annual average census tract populations, population denominators by HPI decile were constructed for mortality rates.^15^ By aggregating cases and population to census tracts and then aggregating and stratifying cases and populations by HPI deciles, we created mortality rates by HPI deciles.

### Covariates

Age categories (0-17, 18-44, 45-64, 65-74, ≥75 years) were used to stratify and age-adjust mortality rates. Sex on death certificates and ACS were limited to included male and female. Death certificates recorded up to 3 races (American Indian/Alaskan Native, Asian, Black, Native Hawaiian/Pacific Islander, other race, two or more races, and White) and the Hispanic ethnicity. Mutually exclusive population subgroups by age, sex, and race/ethnicity were available only for non-Hispanic White and Hispanic people in both the ACS and on death certificates. Subgroups by age, sex, and single race (inclusive of Hispanic) were available for American Indian/Alaskan Native alone (AIAN), Black alone, Native Hawaiian/Pacific Islander alone (NHPI), and two or more races in the ACS and on death certificates. Race-alone classifications of AIAN, other race, and two or more races included a large proportion of Hispanic ethnicity: 53.7%, 98.2%, and 38.2%, respectively.^16^ Other race alone was subsumed in the Hispanic ethnicity and was not used. Analyses of California regions and disaggregated subgroups for Asian, Hispanic, NHPI, and AIAN individuals are beyond the scope of this article and are reported elsewhere.^7^

### Statistical analyses

Mortality rates were age adjusted and covariate stratified by HPI decile. Age adjustment used 5 groups (0-17, 18-44, 45-64, 65-74, and ≥75 years) and their respective 2000 US standard weights (0.257736, 0.393797, 0.222081, 0.066037, and 0.060349).^17^ We calculated exact Poisson 95% CIs for stratum-specific and age-adjusted mortality rates^18^^,^^19^ and mortality rate ratios (RRs).^19^^,^^20^ We calculated the CI for the difference (RD) between 2 age-adjusted mortality rates as ${\mathrm{RD}}_{95\%\mathrm{CI}}=\mathrm{RD}\pm 1.96\times \sqrt{{\mathrm{se}}_{\mathrm{i}}^2+{\mathrm{se}}_{\mathrm{ref}}^2\ }$, where *se* is the standard error of an adjusted rate in the *í*th comparison group and reference group (ref), respectively.^19^

To explore the functional form of the HPI decile-mortality gradient, we used segmental (joinpoint) regression^21^ using log-linear, age-adjusted, or age-stratified rates with corresponding SEs for models with 0, 1, and 2 joinpoints. If non-zero slopes of adjacent segments were not significantly different from each other, we chose a more parsimonious model, including 0 joinpoints. Slopes of line segments were expressed as the average HPI decile percent change (DPC) using the formula $APC={e}^{\beta }-1$, where β was the slope of the log-linear regression. We examined effect modification of the HPI decile-mortality gradient by covariates by testing pairwise combinations of a stratifying variable for parallel slopes.

We set a *P* value of <0.05 (2-sided) for statistical significance. We recognize that in a large data set, small differences in rates or slopes are likely to be statistically significant. Thus, we applied the criterion of practical importance for public health actions. We also recognize that multiple combinations of stratified covariates create a large number of comparisons in which false-positive declarations of statistical significance may occur. We minimized multiple comparisons by planning but did not apply a correction to *P* values. Analyses and data quality control were done in the R statistical package (version 4.2.2),^22^ and segmental regression was carried out in Joinpoint statistical software.^21^ All associations in tables and figures were statistically significant (*P* < 0.05, 2-sided), unless otherwise indicated.

The research protocol for this project was reviewed and approved by the California Health and Human Services Agency’s Committee for the Protection of Human Subjects.

## Results

### Response and description of the population

Of the approximately 1.98 million deaths among California residents between 2015 and 2021, 94.6% had a census tract geocoded from their death certificate. This included California residents who died out of state. Decedents from unknown or HPI-ineligible census tracts tended to be younger, proportionately male and Native American/Alaskan Native, more likely to have lived in a rural county, lack a residential street number or address or have an address descriptor of probable homelessness,^23^ and to have died of injuries or ill-defined causes. Nearly all (98.8%) of the California population reside in HPI-eligible census tracts. The HPI scores were positively associated with older ages and White and Asian populations and lower HPI scores were associated with Black and Latino populations (Table 2). Within race/ethnicity groups, approximately 11% of White and Asian populations resided in HPI quartile 1 census tracts. In contrast, 30% or greater of Black, Asian, and AIAN individuals resided in HPI quartile 1 census tracts.

**Table 2:** Association of Health Places Index population quartiles with age, sex, and race/ethnicity, 2015-2019, California.

	**Quartile of the Healthy Places Index**							
**Item**	**1 (Least healthy community conditions)**		**2**		**3**		**4**	
	**Population**	**%**	**Population**	**%**	**Population**	**%**	**Population**	**%**
Total	9 413 387	100.0	9 909 107	100.0	10 065 532	100.0	9 405 020	100.0
Sex								
Male	4 712 148	50.1	4 895 917	49.4	4 964 877	49.3	4 632 777	49.3
Female	4 701 239	49.9	5 013 190	50.6	5 100 655	50.7	4 772 243	50.7
Age in years								
<18	2 639 606	28.0	2 313 518	23.3	2 118 777	21.0	1 913 120	20.3
18-44	3 804 031	40.4	3 871 491	39.1	3 778 694	37.5	3 193 137	34.0
45-64	2 009 391	21.3	2 384 807	24.1	2 630 994	26.1	2 689 571	28.6
65-74	562 977	6.0	769 367	7.8	890 934	8.9	923 782	9.8
≥75	397 382	4.2	569 924	5.8	646 133	6.4	685 410	7.3
Race/ethnicity								
Non-Hispanic White	1 604 094	17.0	3 026 709	30.5	4 451 757	44.2	5 337 271	56.7
Non-Hispanic Black	733 306	7.8	611 920	6.2	504 317	5.0	260 137	2.8
Non-Hispanic AIAN	41 825	0.4	41 851	0.4	34 599	0.3	17 796	0.2
Non-Hispanic Asian	606 491	6.4	1 219 129	12.3	1 724 888	17.1	1 998 576	21.3
Non-Hispanic NHPI	30 267	0.3	39 539	0.4	44 758	0.4	24 160	0.3
Non-Hispanic Other	19 000	0.2	25 557	0.3	26 327	0.3	28 144	0.3
Multirace, any Hispanic	332 385	3.5	469 618	4.7	564 693	5.6	526 861	5.6
Hispanic, single race	6 046 019	64.2	4 474 784	45.2	2 714 193	27.0	1 212 075	12.9

Abbreviations: AIAN, American Indian/Alaskan Native; NHPI, Native Hawaiian/Pacific Islander.

On average, compared with the first HPI decile, people living in 10th decile had more than 5 times the per capita income and were more likely to be employed, live above poverty level, be college educated, have young children enrolled in preschool, participate in voting and the decennial Census, have health insurance and access to a motor vehicle, and commute to work by cycling, walking, or transit (Table 1). They tended to own their home, experience less overcrowding and housing cost burden, and live in neighborhoods with more tree canopy, nearby parks, more retail establishments, less ambient air pollution, and higher drinking water quality. Of note, based on historical maps of the Home Owner’s Loan Corporation for 8 major California cities,^24^ 96.8% of areas comprising the first decile and 50% of 10^th^ decile census tracts were not eligible for federally financed home mortgages (ie, they were “redlined”) in 1935-1940 (a C or D rating).

### Social gradient

**Figure 1:**
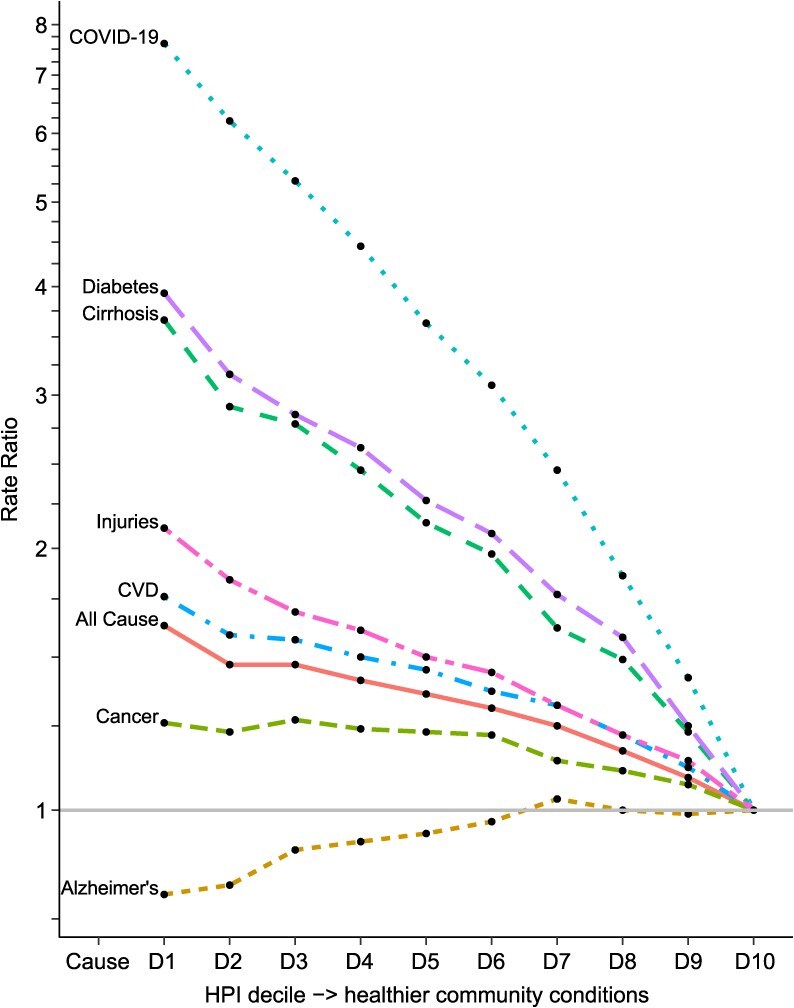
Age-adjusted rate ratios of selected leading causes of death by decile (D) of the Healthy Places Index (10th decile reference), 2015-2019, and COVID-19 (2020-2021), California. Global Burden of Disease Codes: malignant neoplasms, B.01-B.17, B.99; cardiovascular disease (CVD), including stroke: C.01-C.05, C.99; Alzheimer’s disease and other dementias: D.06; cirrhosis of the liver: J.2; diabetes: D.01; injuries: E.01-E.08, E.99.

Age-adjusted mortality rates and RRs decreased monotonically with increasing HPI deciles for all causes combined, cardiovascular disease, cancers, cirrhosis of the liver, diabetes, injuries, and COVID-19 (Tables 3 and 4, Figure 1). Mortality rates for COVID-19, injuries, diabetes, and cirrhosis had the steepest decline (DPC of −7% to −18.5%; *P* < 0.05). For cardiovascular disease, cancer, and COVID-19, the rate of decline accelerated after the sixth or seventh decile. For all causes, the ratio of age-adjusted mortality rates of the 1st and 10th (RR_D1/D10_) HPI deciles was 1.63 (95% CI, 1.60-1.66). There were 60 852 attributable deaths, or 24% of the annual average of 253 730 deaths in 2015-2019 (Table 5). For COVID-19 deaths, the RR_D1/D10_ was 7.61 (95% CI, 7.14-8.12). In 2020-2021, there were an annual average of 25 779 attributable COVID-19 deaths (72%). The RR_D1/D10_ was more than double for diabetes (3.93; 95% CI, 3.42-4.53), cirrhosis of the liver (3.66; 95% CI, 3.21-4.17), and injuries (2.11; 95% CI, 1.97-2.27). The HPI population attributable fractions for diabetes and cirrhosis of the liver were greater than 50% (Table 5).

**Table 3:** Association of age-adjusted annual California mortality rates per 100 000 and rate ratios by HPI decile, all causes of death and COVID-19.

**HPI decile**	**All causes, 2015-2019**			**COVID-19, 2020-2021**		
	**Deaths**	**Rate (95% CI)**	**RR (95% CI)**	**Deaths**	**Rate (95% CI)**	**RR (95% CI)**
1	21 618	789 (778-800)	1.63 (1.60-1.66)	5440	196 (190-201)	7.61 (7.14-8.12)
2	23 286	714 (705-724)	1.47 (1.45-1.50)	5308	159 (155-164)	6.20 (5.81-6.61)
3	25 080	711 (702-719)	1.47 (1.44-1.49)	4928	136 (132-140)	5.29 (4.96-5.65)
4	27 286	682 (673-690)	1.41 (1.38-1.43)	4655	114 (111-118)	4.45 (4.17-4.75)
5	28 098	659 (651-667)	1.36 (1.33-1.38)	4038	93 (90-96)	3.63 (3.39-3.88)
6	28 031	634 (627-642)	1.31 (1.29-1.33)	3570	79 (77-82)	3.08 (2.88-3.30)
7	27 217	607 (599-614)	1.25 (1.23-1.27)	2884	63 (61-66)	2.46 (2.30-2.64)
8	25 899	566 (559-573)	1.17 (1.15-1.19)	2216	48 (46-50)	1.86 (1.73-2.00)
9	25 365	530 (524-537)	1.09 (1.07-1.11)	1778	37 (35-38)	1.42 (1.32-1.54)
10 (Ref.)	21 850	485 (478-491)	1.00	1168	26 (24-27)	1.00
DPC^a^		–4.7			–12.3	

Abbreviations: DPC, decile percent change; HPI, Healthy Places Index; RR, rate ratio.

a All DPCs are statistically significant (*P* < 0.05); no joinpoints were detected.

**Table 4:** Association of age-adjusted annual California mortality rates per 100 000 and rate ratios by Healthy Place Index decile for selected causes of death^a^, 2015-2019.

**HPI decile**	**Cardiovascular diseases**			**Malignant neoplasms**			**Alzheimer’s disease**		
	**No. of deaths**	**Rate (95% CI)**	**RR (95% CI)**	**No. of deaths**	**Rate (95% CI)**	**RR (95% CI)**	**No. of deaths**	**Rate (95% CI)**	**RR (95% CI)**
1	6934	267 (261-274)	1.76 (1.71-1.83)	4118	149 (145-154)	1.26 (1.21-1.31)	1184	53 (50-56)	0.80 (0.75-0.86)
2	7638	241 (236-247)	1.59 (1.54-1.65)	4843	146 (142-150)	1.23 (1.18-1.28)	1552	54 (51-56)	0.82 (0.77-0.87)
3	8219	238 (233-243)	1.57 (1.52-1.62)	5427	150 (146-155)	1.27 (1.22-1.31)	1875	59 (56-61)	0.90 (0.84-0.95)
4	9014	228 (223-232)	1.50 (1.46-1.55)	6032	147 (143-151)	1.24 (1.19-1.28)	2280	60 (58-63)	0.92 (0.87-0.97)
5	9384	220 (216-225)	1.45 (1.41-1.50)	6368	146 (143-150)	1.23 (1.19-1.27)	2561	62 (59-64)	0.94 (0.89-0.99)
6	9116	207 (203-212)	1.37 (1.33-1.41)	6567	144 (141-148)	1.22 (1.17-1.26)	2692	63 (61-66)	0.97 (0.92-1.02)
7	8940	200 (196-204)	1.32 (1.28-1.36)	6290	136 (132-139)	1.14 (1.10-1.18)	2930	67 (65-70)	1.03 (0.98-1.09)
8	8468	185 (181-189)	1.22 (1.18-1.26)	6237	132 (129-135)	1.11 (1.07-1.15)	2926	65 (63-68)	1.00 (0.95-1.05)
9	8155	169 (166-173)	1.12 (1.08-1.16)	6276	128 (124-131)	1.07 (1.04-1.11)	3068	65 (62-67)	0.99 (0.94-1.04)
10 (Ref.)	6837	151 (148-155)	1.00	5533	119 (116-122)	1.00	2909	65 (63-68)	1.00
DPC^b^		–5.2			–2.4			2.3	
Joinpoints: DPC^b^		1-7: –4.2			1-6: 0.7^c^			1-7: 2.3	
		7-10: –8.5			6-10: –4.6			7-10: –0.6^c^	
	**Cirrhosis of the liver**			**Diabetes**			**Injuries**		
	**No. of deaths**	**Rate (95% CI)**	**RR (95% CI)**	**No. of deaths**	**Rate (95% CI)**	**RR (95% CI)**	**No. of deaths**	**Rate (95% CI)**	**RR (95% CI)**
1	841	27 (25-29)	3.66 (3.21-4.17)	695	26 (24-28)	3.93 (3.42-4.53)	2294	67 (64-70)	2.11 (1.97-2.27)
2	773	22 (20-23)	2.91 (2.55-3.32)	676	21 (19-22)	3.17 (2.75-3.65)	2153	59 (56-61)	1.84 (1.72-1.98)
3	796	21 (19-22)	2.78 (2.44-3.17)	664	19 (17-20)	2.85 (2.48-3.28)	2063	54 (51-56)	1.69 (1.57-1.82)
4	775	18 (17-20)	2.46 (2.16-2.81)	690	17 (16-18)	2.61 (2.27-3.00)	2095	51 (49-53)	1.61 (1.50-1.73)
5	698	16 (15-17)	2.14 (1.88-2.45)	638	15 (14-16)	2.27 (1.97-2.62)	1961	48 (46-50)	1.50 (1.40-1.62)
6	677	15 (14-16)	1.97 (1.72-2.25)	611	14 (12-15)	2.08 (1.80-2.40)	1966	46 (44-48)	1.44 (1.34-1.55)
7	561	12 (11-13)	1.62 (1.41-1.86)	527	11 (11-13)	1.77 (1.53-2.05)	1803	42 (40-44)	1.32 (1.23-1.42)
8	521	11 (10-12)	1.49 (1.29-1.71)	479	10 (9-11)	1.58 (1.36-1.84)	1653	39 (37-41)	1.22 (1.13-1.31)
9	441	9 (8-10)	1.23 (1.06-1.43)	394	8 (7-9)	1.25 (1.07-1.46)	1530	36 (34-38)	1.14 (1.05-1.23)
10 (Ref.)	340	7 (7-8)	1.00	300	7 (6-7)	1.00	1279	32 (30-34)	1.00
DPC^b^		–12.3			–12.6			2.3	
Joinpoints: DPC^b^		None			None				
								1-7: 2.3	
								7-10: –0.6^c^	

Abbreviations: DPC, decile percent change; HPI, Healthy Places Index; RR, rate ratio.

a Global Burden of Disease Codes: malignant neoplasms, B.01-B.17, B.99; cardiovascular disease, including stroke: C.01-C.05, C.99; Alzheimer’s disease and other dementias: D.06; cirrhosis of the liver: J.2; diabetes: D.01; injuries: E.01-E.08, E.99.

b All DPCs are statistically significant (*P* < 0.05), unless noted by a superscript “c.”

**Table 5:** Annual Healthy Places Index–attributable deaths and population attributable fractions for major causes and COVID-19, California.^a^

**Cause**	**No. of attributable deaths**	**Population attributable fraction**
All causes (2015-2019)	60 852	0.24
Cardiovascular disease, including stroke	23 149	0.28
Malignant neoplasms	9055	0.17
Injuries	6262	0.33
Cirrhosis	3316	0.52
Diabetes	3037	0.54
COVID-19 (2020-2021)	25 779	0.72

a The number of deaths that could be avoided if the entire California population experienced the lower mortality rates of people residing in the Healthy Places Index decile 10. Mortality rates for Alzheimer’s disease and related disorders did not show a consistent inverse gradient with respect to the Healthy Places Index.

### Effect modification

**Figure 2:**
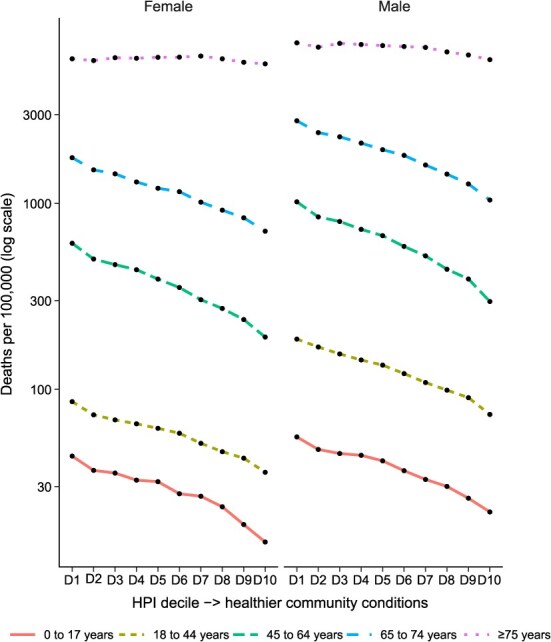
Age-specific, 5-year annual average all causes mortality rate (per 100 000) by decile (D) of the Healthy Places Index and sex, California, 2015-2019.

Within each age stratum, all-cause mortality rates monotonically decreased with increasing HPI decile (Table 6, Figure 2). The strength of association was greatest in ages 45 to 64 years (RR_D1/D10_: 3.34) and weakest in those aged 75 years or older (RR_D1/D10_: 1.13). In pairwise comparisons among the 5 age strata of HPI decile-mortality gradients, parallel slopes were rejected between each of the 4 youngest age groups and the oldest. Age-stratified COVID-19 mortality rates demonstrated a steep gradient across HPI deciles, particularly among those younger than 75 years (Table 7). For ages 18 to 44 years and 45 to 64 years, COVID-19 mortality rates in the first HPI decile were more than 20 times that of 10th. For each age group, joinpoints were found at decile 6 or 7, after which COVID-19 mortality rates decreased more steeply. In pairwise comparisons among the 4 oldest age strata, parallel slopes were rejected between each of the 3 age groups aged less than 75 years and 75 years or older. Cardiovascular disease, cirrhosis, and diabetes had inverse HPI-mortality gradients in each age stratum, including 75 years or older (DPC of −2.27, −8.10, and −9.98, respectively). Alzheimer’s disease, cancer, and injuries had inverse HPI-mortality gradients in age groups younger than 75 years, but a positive association with HPI deciles at age 75 or older (DPC of 2.56, 0.29, and 1.13, respectively).

**Table 6:** Age-stratified, annual all-causes mortality rates per 100 000 by Healthy Places Index decile, California, 2015-2019.

	**Age, years**									
	**0–17**		**18–44**		**45–64**		**65–74**		**≥75**	
	**No. of deaths**	**Rate (95% CI)**	**No. of deaths**	**Rate (95% CI)**	**No. of deaths**	**Rate (95% CI)**	**No. of deaths**	**Rate (95% CI)**	**No. of deaths**	**Rate (95% CI)**
HPI decile										
1	567	50 (46-54)	2100	138 (132-144)	6003	812 (792-833)	4319	2237 (2171-2305)	8629	6520 (6383-6658)
2	426	42 (38-46)	1863	122 (116-127)	5611	671 (653-688)	4602	1924 (1869-1980)	10 785	6286 (6168-6405)
3	403	40 (37-45)	1741	112 (107-118)	5637	630 (614-647)	4865	1827 (1776-1878)	12 434	6548 (6434-6664)
4	366	38 (35-43)	1653	105 (100-110)	5586	580 (565-595)	5077	1673 (1627-1719)	14 603	6477 (6373-6583)
5	315	37 (33-41)	1487	99 (94-104)	5062	526 (512-541)	5106	1543 (1501-1586)	16 128	6490 (6391-6591)
6	288	32 (29-36)	1403	90 (86-95)	4910	467 (454-480)	5042	1460 (1420-1501)	16 388	6468 (6370-6568)
7	246	30 (26-34)	1213	81 (76-85)	4299	409 (397-422)	4669	1288 (1252-1326)	16 790	6482 (6384-6580)
8	211	27 (23-31)	1037	73 (69-77)	3811	355 (344-367)	4226	1158 (1124-1193)	16 615	6212 (6118-6307)
9	173	22 (19-26)	870	67 (62-71)	3400	313 (303-324)	3923	1042 (1010-1075)	17 000	5966 (5877-6056)
10	139	19 (16-22)	642	54 (50-59)	2574	243 (234-253)	3164	867 (837-897)	15 330	5757 (5667-5849)
DPC^a^		–8.9		–8.7		–11.2		–9.1		–1.2
Joinpoint: DPC		None		None		None		None		1-7:0.1
										7-10: –4.0
	**RR**	**95% CI**	**RR**	**95% CI**	**RR**	**95% CI**	**RR**	**95% CI**	**RR**	**95% CI**
1	2.69	2.23-3.23	2.53	2.32-2.76	3.34	3.19-3.50	2.58	2.47-2.70	1.13	1.10-1.16
2	2.28	1.88-2.76	2.24	2.05-2.45	2.76	2.63-2.89	2.22	2.12-2.32	1.09	1.07-1.12
3	2.18	1.80-2.65	2.06	1.89-2.26	2.59	2.47-2.72	2.11	2.02-2.20	1.14	1.11-1.16
4	2.07	1.71-2.52	1.93	1.76-2.12	2.39	2.28-2.50	1.93	1.85-2.02	1.13	1.10-1.15
5	1.98	1.62-2.41	1.81	1.65-1.99	2.16	2.06-2.27	1.78	1.70-1.86	1.13	1.10-1.15
6	1.73	1.41-2.12	1.66	1.51-1.82	1.92	1.83-2.02	1.68	1.61-1.76	1.12	1.10-1.15
7	1.61	1.31-1.98	1.48	1.34-1.63	1.68	1.60-1.77	1.49	1.42-1.55	1.13	1.10-1.15
8	1.44	1.16-1.79	1.34	1.21-1.48	1.46	1.39-1.54	1.34	1.28-1.40	1.08	1.06-1.10
9	1.21	0.97-1.52	1.22	1.10-1.35	1.29	1.22-1.36	1.20	1.15-1.26	1.04	1.01-1.06
10 (Ref.)	1.00 (reference)		1.00		1.00		1.00		1.00	

Abbreviations: DPC, decile percent change; HPI, Healthy Places Index; RR, rate ratio.

a *P* < .05 for all DPC values.

**Table 7:** Age-stratified^a^, annual COVID-19 mortality rates per 100 000 by HPI decile, California, 2020-2021.

**HPI decile**	**18 to 44 Years**		**45 to 64 Years**		**65 to 74 Years**		**75+ Years**	
	**Deaths**	**Rate (95% CI)**	**Deaths**	**Rate (95% CI)**	**Deaths**	**Rate (95% CI)**	**Deaths**	**Rate (95% CI)**
1	414	27 (25-30)	1790	242 (231-254)	1426	738 (701-777)	1807	1365 (1303-1429)
2	335	22 (20-24)	1611	193 (183-202)	1345	562 (533-593)	2014	1174 (1123-1226)
3	281	18 (16-20)	1437	161 (152-169)	1248	468 (443-495)	1961	1033 (988-1079)
4	246	16 (14-18)	1204	125 (118-132)	1068	352 (331-374)	2135	947 (907-988)
5	186	12 (11-14)	944	98 (92-105)	929	281 (263-299)	1978	796 (761-831)
6	161	10 (9-12)	816	78 (72-83)	803	232 (217-249)	1790	706 (674-740)
7	114	8 (6-9)	571	54 (50-59)	592	163 (150-177)	1605	620 (590-651)
8	69	5 (4-6)	415	39 (35-42)	410	112 (102-124)	1322	494 (468-521)
9	27	2 (1-3)	259	24 (21-27)	299	79 (71-89)	1194	419 (396-443)
10	15	1 (1-2)	118	11 (9-13)	166	45 (39-53)	868	326 (305-348)
DPC^b^		–20.9		–23.6		–23.5		–13.8
Joinpoint: DPC		1-7: –18.0		1-7: –20.8		1-6: –20.6		1-7: –12.2
		7-10: –46.8		7-10: –39.7		6-10: –31.0		7-10: –22.6
	**RR**	**95% CI**	**RR**	**95% CI**	**RR**	**95% CI**	**RR**	**95% CI**
1	21.3	12.7-35.7	21.8	18.1-26.3	16.3	13.9-19.1	4.2	3.9-4.5
2	17.2	10.3-28.9	17.4	14.4-20.9	12.4	10.6-14.6	3.6	3.3-3.9
3	14.2	8.5-24.0	14.5	12.0-17.5	10.3	8.8-12.2	3.2	2.9-3.4
4	12.3	7.3-20.7	11.3	9.3-13.6	7.8	6.6-9.1	2.9	2.7-3.1
5	9.7	5.7-16.4	8.8	7.3-10.7	6.2	5.3-7.3	2.4	2.3-2.7
6	8.1	4.8-13.8	7.0	5.8-8.5	5.1	4.3-6.1	2.2	2.0-2.4
7	5.9	3.5-10.2	4.9	4.0-6.0	3.6	3.0-4.3	1.9	1.8-2.1
8	3.8	2.2-6.6	3.5	2.8-4.3	2.5	2.1-3.0	1.5	1.4-1.7
9	1.6	0.9-3.0	2.2	1.7-2.7	1.8	1.5-2.1	1.3	1.2-1.4
10 (Ref.)	1.0		1.0		1.0		1.0	

Abbreviations: DPC, decile percent change; HPI, Healthy Places Index; RR, rate ratio.

a Mortality rates of those aged 0-17 years were not statistically reliable (relative SE > 0.5) and were excluded.

b All DPC values *P* < 0.05.

In each age and sex stratum (Figure 2), all-cause mortality rates declined monotonically with increasing HPI decile. At each HPI decile, men had higher mortality rates than women. In joinpoint regression, parallel slopes were rejected for the male and female all-cause mortality gradients. The sex-stratified, age-adjusted COVID-19 mortality–HPI gradient followed the same pattern as all-cause mortality, with the salient difference that the strength of association was many times greater: an RR_D1/D10_ in women of 6.85 (95% CI, 6.21-7.56) and in men of 8.25 (95% CI, 7.56-9.00).

**Figure 3:**
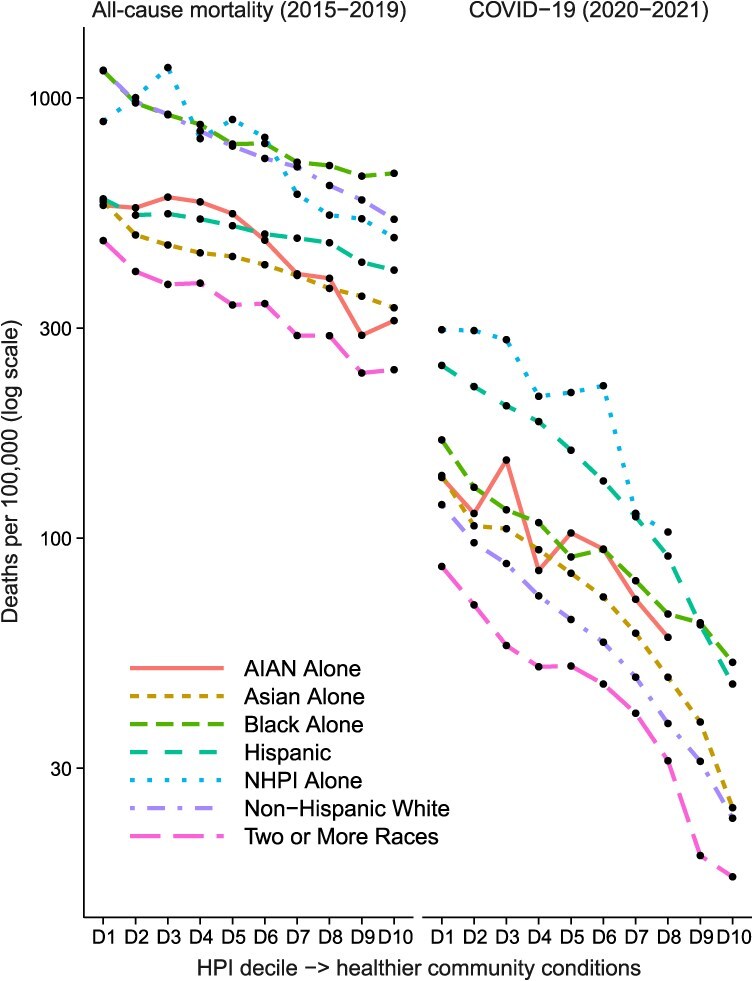
Annual age-adjusted California mortality rates by race/ethnicity and decile (D) of the Healthy Places Index (HPI), all causes (2015-2019) and COVID-19 (2020-2021). COVID-19 mortality rates in HPI deciles 9 and 10 for American Indian/Alaskan Native alone (AIAN) alone and Native Hawaiian/Pacific Islander alone (NHPI) alone are not shown, to comply with state of California data de-identification guidelines.

In age-adjusted, race/ethnicity-stratified analyses of all-cause mortality (Figure 3), each race/ethnicity group tended toward lower mortality rates with increasing HPI decile. The average DPC in the HPI-mortality gradient was steepest for the two or more races category (−23.5%), followed by Asian alone and non-Hispanic White (−14.9%), NHPI alone and AIAN alone (−14.9%), Hispanic (−13.8%), and Black alone (−9.2%). At each decile, Black alone, non-Hispanic White, and NHPI alone had higher all-cause mortality rates than Hispanic, Asian alone, AIAN alone, and the two or more races category. Joinpoints were identified at the fourth decile for AIAN alone and the third decile for NHPI alone. Slopes for the first segment were nearly level, after which rates declined at 11.5% per decile for AIAN alone and 11.7% per decile for NHPI alone. For the other race/ethnicity groups, no joinpoints were identified.

For age-adjusted all-cause mortality rates, differences between the highest and lowest HPI deciles within race/ethnicity groups were often larger than differences between Asian alone individuals (reference) and other race/ethnicity groups (Table 8). For example, the difference in age-adjusted mortality rates between Asian alone and non-Hispanic White individuals was 312 per 100 000. This difference was smaller than that between the 1st and 10th deciles within non-Hispanic White individuals (626 per 100 000). Similar findings were observed for COVID-19 but with non-Hispanic White mortality rates serving as the race/ethnicity reference (Table 8).

**Table 8:** Differences in annual age-adjusted all causes mortality rates between HPI deciles 1 and 10 and between race/ethnicity groups, California, 2015-2019.

	**All causes, 2015-2019**			**COVID-19, 2020-2021**		
	**Between Asian alone and other race/ethnicity groups**		**Between HPI decile 1-10 within race/ethnicity groups**	**Between non-Hispanic White and other race/ethnicity groups**		**Between HPI decile 1-10 within race/ethnicity groups**
**Group**	**Rate ratio**	**Rate difference per 100 000 (95% CI)**	**Rate difference per 100 000 (95% CI)**	**Rate ratio**	**Rate difference per 100 000 (95% CI)**	**Rate difference per 100 000 (95% CI)**
Total			304 (291-317)			170 (164-176)
Asian alone^a^	1.00	0 (reference)	248 (213-284)	1.26	14 (0-29)	114 (97-131)
AIAN alone	1.24	97 (69-125)	258 (91-425)	1.77	41 (28-53)	57 (0-160)
Black alone	2.16	462 (449-475)	478 (411-546)	2.00	53 (46-59)	115 (91-139)
Hispanic	1.31	123 (116-130)	184 (156-211)	3.28	120 (114-125)	200 (188-212)
NHPI alone	1.86	343 (294-392)	402 (102-703)	3.28	119 (96-142)	215 (37-392)
NH White^b^	1.78	312 (306-318)	626 (597-654)	1.00	0 (reference)	96 (87-105)

Abbreviations: AIAN, American Indian/Alaskan Native; HPI, Healthy Places Index; NH, non-Hispanic; NHPI, Native Hawaiian/Pacific Islander.

a The Asian alone age-adjusted annual all-cause mortality rate was the lowest (399 per 100 000) among race/ethnicity groups.

b The non-Hispanic White age-adjusted annual COVID-19 mortality rate was the lowest (119 per 100 000) among race/ethnicity groups.

Each race/ethnicity group experienced steep declines in COVID-19 deaths with increasing HPI decile (Figure 3). The NHPI alone, Hispanic, and Black alone categories generally experienced the highest mortality rates across HPI deciles. The Asian alone, Hispanic, non-Hispanic White, and two or more races categories each experienced an RR_D1/D10_ exceeding 5.

## Discussion

We demonstrated an inverse association between the HPI and death due to all causes, most leading causes (prepandemic), and COVID-19. The steepness of the gradient was modulated by specific cause, and by age, sex, and race/ethnicity within causes. The steepness of the gradient and attributable fraction was pronounced for several causes, including injures, diabetes, cirrhosis of the liver, and, most notably, COVID-19.

The contrast between prepandemic deaths and COVID-19 includes a remarkable reordering of race/ethnicity-specific mortality rates that highlights the interplay between SDOH and race/ethnicity in generating health inequities. For all and major prepandemic causes, Latino individuals had lower mortality rates than did White and Black individuals. This is consistent with widely reported disparities attributed to population selection related to immigration, acculturation, and social capital (eg, “healthy immigrant effect”, “Latino paradox).^25^ However, during the first 2 years of the COVID-19 pandemic, the historical mortality advantage of Latino populations was reversed and non-Hispanic White individuals experienced lower COVID-19 mortality rates. The steepness of the COVID-19 gradient, its racial order, and the enormous disparity among the young working-age population (RR > 20) appear to be pathognomic of a highly transmissible and lethal virus with access to a racially stratified, essential workforce whose socioeconomic profile overlapped the lowest HPI deciles,^26^ a general population with a preexisting gradient in the distribution of SDOH, and a breakdown of upstream systems for ensuring healthy and safe work,^27^ housing, transportation, public health, and health care.^28^ Several HPI indicators closely link to pathways of virus transmission, but they may also contribute to the allostatic load that associates SDOH with chronic disease.^29^

Besides monitoring health inequity during the pandemic, the HPI guided interventions for local health jurisdictions and health care systems. State health agencies directed local health jurisdictions to create targeted investment plans that funded $272 million in educational outreach and support services.^30^ Nearly all county plans used HPI maps to geographically pinpoint priority communities and community partners.^5^ Private sector health organizations used HPI to prioritize and deploy mobile vaccination units and community outreach.^31^ During the initial rollout of vaccine distribution, the disproportionate proportion of COVID-19 deaths (40% of deaths in the first HPI quartile) was used by California health agencies to justify the allocation of 40% of available vaccines for HPI quartile 1 communities. An evaluation of this equity enhancement reported that in the succeeding 8 months (March-October 2021), California averted more than 160 800 COVID-19 cases, 10 200 hospitalizations, and 675 deaths.^32^ The generalizability of this strategy and its impact beyond California may depend on state-specific COVID-19 public health responses that varied in timing and intensity.^33^

Despite differences in international settings, socioeconomic indexes, time periods, and areal units, there is remarkable consistency in the social gradient in this study and those reported over the past 3 decades in England and Wales,^34^^-^^36^ Scotland,^37^ Canada,^38^ Australia,^39^^,^^40^ and the United States.^41^^,^^42^ This is not surprising, given the overlap of domains and indicators among indexes. The findings are consistent with a large body of research on death and income,^43^ education, and occupation.^44^^-^^49^

Aggregations of areal units overlap with “areal policies” that determine the distribution of social determinants of health. For example, land use decisions are made by local planning agencies. Single-family zoning,^50^ together with redlining are areal policies that enforced racial residential segregation and diminished wealth creation and its intergenerational transmission in Black and other non-White communities. It is interesting to note that the Black-White disparity in all-cause mortality (Figure 3) was not apparent until after the third HPI decile and became increasingly pronounced at higher deciles, consistent with the well-documented Black-White wealth gap and adverse health impacts.^51^^,^^52^ In the transportation sector, urban renewal projects and eminent domain for urban highway and other transportation projects leveled whole communities and displaced their residents. Underfunding of public transportation infrastructure and service, particularly in low-income and communities of color, is well documented,^53^ as is community severance in highway projects. Exposures to air, water, and soil contaminants also reflect areal policies related to siting of hazardous waste sites and toxic air emitters, which have been documented in the environmental justice literature^54^ and echo HPI’s environmental indicators (Table 1).

To explain the mortality gradient, researchers point to higher prevalence of behavioral risk factors in the most deprived in multiple deprivation indexes.^55^ We also observed a strong inverse pattern in the prevalence of low physical activity, current smoking, and obesity in adults by HPI decile (prevalence RR_D1/D10_ of 2.48, 2.37, and 1.80, respectively).^56^ However, personal health behaviors are intervening distal variables in a causal chain set in motion by upstream “economic policies and systems, development agendas, social norms, social policies and political systems.”^57^^-^^59^

### Strengths and weaknesses

The large California population and several years of mortality data produced a sample that facilitated statistically reliable results and the control of confounding. A large sample also enabled us to explore effect modification by age, sex, and race/ethnicity, and the functional form of the social gradient.

Compared with individual-level variables similarly defined, the strength of association with health outcomes is underestimated using area-based socioeconomic measures, and the degree of underestimation increases with the size of the areal unit.^60^^,^^61^ Except for unique research settings that have access to individual level data,^49^ area-based measures yield reliable but conservative estimates of social gradients, and represent the best, if not only, option to monitor health inequities for routine public health surveillance.

Cross-sectional study designs are inherently limited. Decedents were assigned an HPI score based on residence at time of death, which does not consider change over the life cycle. Duration of deprivation was not available and may align with prevailing hypotheses regarding allostatic load, weathering, and cumulative psychosocial stressors.^29^ We also did not have data on the timing of exposure to unhealthy community conditions and latency of health events, the importance of which has been shown in adverse childhood experiences studies.^62^ Examining pre– and post–COVID-19 changes in mortality rates by cause was beyond the scope of this study.

A small percentage of decedents had missing census tract in death data; this disproportionately affected AIAN decedents. Misclassification of race/ethnicity on death certificates is well documented,^63^^,^^64^ particularly underestimating AIAN and NHPI deaths. We do not know whether these were differentially expressed across HPI deciles. Specific causes of death may be undercounted or misclassified as the underlying cause of death on death certificates and may bias association toward the null if randomly associated with exposure. In the case of Alzheimer’s disease, biases in diagnosis of Alzheimer’s disease by race/ethnicity^65^ and neighborhood disadvantage^66^ have been documented and, in cross-sectional studies such as ours, competing causes of death associated with exposure may produce contrary disease-exposure associations.^67^ Because of limitations in the ACS, we were not able to create mutually exclusive race/ethnicity groupings that could be used in age adjustment. “Alone” breakdowns for Black, Asian, AIAN, NHPI, and the two or more races category included Hispanic people, whose generally lower mortality rates also lowered the mortality rates of races with whom they were combined. The burden of traumatic workplace injuries and chronic occupational disease is not attributed in residential place-based indexes.

### Public health implications

If health inequities related to social determinants of health were eliminated in California, a large percentage of deaths would have been averted before and during the COVID-19 pandemic. Our findings confirm that social determinants of health are a leading cause of “preventable and unfair” death in California. To our knowledge, ours is the first study of its kind in California.

Our findings reinforce the observation that the dose-response function of death and SDOH is a gradient covering the entire population. The implications are that interventions that only focus on the poor (eg, first 2 HPI deciles) will not address the roughly 65% of HPI-attributable deaths that occur between the 20th and 80th decile (“middle class”). Interventions that are proportional to the magnitude of the disease burden, as suggested by Marmot et al.,^68^ may best fit the population dynamics of SDOH. Many potential upstream and downstream interventions and empowerment strategies to address racial and social class health disparities have been catalogued by us^69^ and others,^68^^,^^70^ and they scale from local to societally transformative. We also showed that SDOH-related health disparities within race/ethnicity groups can be as large as those between race/ethnicity groups. Thus, interventions to eliminate health disparities ideally should address race, place, and social class. Last, California, like the rest of the United States, does not have an SDOH-based health equity component to its routine public health surveillance systems. In the immediate future, it is not likely that individual-level SDOH will be collected in public health surveillance systems. This study illustrates how routine mortality surveillance can push beyond age, sex, race/ethnicity, and urban/rural as determinants of health and include measures of social class. This adds essential information for public health policymaking and evaluation, and, when linked to action, contributes to the goals of improving health and health equity.

## Data Availability

State of California data de-identification guidelines prohibit public release of potentially re-identifiable disaggregated data of dead individuals. Requests for aggregated tabular data that meet data de-identification guidelines should be directed to the authors.
